# Sensory Acceptability of Multiple-Micronutrient-Fortified Lentils in Bangladesh

**DOI:** 10.3390/foods13244081

**Published:** 2024-12-17

**Authors:** Rajib Podder, Fakir Md Yunus, Nurjahan Binte Munaf, Farzana Rahman, Fouzia Khanam, Mohammad Delwer Hossain Hawlader, Albert Vandenberg

**Affiliations:** 1 Department of Plant Sciences, University of Saskatchewan, Saskatoon, SK S7N 5A8, Canada; 2 Department of Psychology and Neuroscience, Dalhousie University, 1355 Oxford St., Halifax, NS B3H 4R2, Canada; fakir.yunus@dal.ca; 3 Department of Public Health, North South University, Dhaka 1229, Bangladesh; nurjahan.binte@northsouth.edu (N.B.M.); farzanarahman9991@gmail.com (F.R.); fouzia.khanam@northsouth.edu (F.K.); mohammad.hawlader@northsouth.edu (M.D.H.H.)

**Keywords:** lentil, fortification, vitamins, minerals, sensory evaluation

## Abstract

In this study, panelists in rural Bangladesh (*n* = 150) assessed the sensory attributes of two cooked and uncooked dehulled red lentils: the control (unfortified lentils) and lentils fortified with eight vitamins and two minerals (multiple micronutrient fortified; MMF). The panelists evaluated the appearance, odor, and overall acceptability using a nine-point hedonic scale (1 = extremely dislike; 9 = extremely like). The taste and texture of the cooked lentils, prepared as South Asian lentil meals, were assessed. Consumer responses varied significantly in the appearance of the uncooked lentils but were similar in odor and overall acceptability. Meanwhile, the five traits of the cooked lentils, including overall acceptability, showed significantly similar consumer responses. This suggests that fortification had a minimal impact on the sensory qualities of the MMF lentils. Furthermore, a highly significant (*p* < 0.0001) correlation coefficient (with values ranging from −0.98 to 0.97) was observed between HunterLab colorimetric measurements (L = luminosity, a* = red hue, and b* = yellow hue) and sensory trait ratings. The Cronbach’s alpha (CA) score for both the cooked control and MMF lentils was 0.79. The average CA score for the cooked lentils was 0.79, while for the uncooked lentils, it was 0.71, demonstrating the strong reliability of the panelists’ assessments. Overall, the sensory qualities of the MMF lentils were acceptable and did not differ significantly from those of the control lentils.

## 1. Introduction

Micronutrient deficiency is a global health issue and remains an economic burden. Micronutrients such as iron (Fe), zinc (Zn), calcium (Ca), vitamin A (retinol and β carotene), thiamin (vitamin B1), riboflavin (vitamin B2), niacin (vitamin B3), folate (vitamin B9), and cyanocobalamin (vitamin B12) are crucial for all living organisms to maintain ideal physiological function. Shortages of these essential nutrients pose a significant global health concern. Also, such deficiencies can lead to inadequate physical and cognitive development in children, increased susceptibility to or worsening of diseases, cognitive disabilities, vision impairment, and reduced overall productivity and potential [1]. Estimates indicate that globally, more than 50% of preschool-aged children and about two-thirds of non-pregnant women of childbearing age experience micronutrient deficiencies [2]. Plant-based diets are gaining popularity worldwide, and legumes like lentils, chickpeas, dry peas, beans, and faba beans play a significant role as dietary protein sources. In many countries, vitamin B12, calcium, folate, and other vitamins and micronutrients are also increasingly important for plant-based foods in vegetarian and vegan diets. Lentils stand out among legumes due to their exceptional protein, carbohydrate, and essential micronutrient contents, which exceed some staple cereals and root vegetables. Collectively, over 50 countries produce approximately 6.65 million tonnes of lentils globally, with Canada contributing about 50% (2.20 million tonnes) of this total [3]. Lentils, as a type of legume, have 25.8–27.1% protein (dry weight), 27.4–47.1% starch,5.1–26.6% dietary fiber, 73–90 mg kg^−1^ of iron, 44 to 54 mg kg^−1^ of zinc, and 425–673 µg kg^−1^ of selenium [4]. Lentils are also abundant in vitamins A, B1, and B9 and β-carotene, and they also offer substantial amounts of vitamins B1, B3, B5, B6, K, and E [5,6]. In many Asian countries, including Bangladesh, a combination of rice and lentils creates a popular dish known as “hotchpotch”. This meal is a nutritional powerhouse, offering almost every vital amino acid, dietary fiber, carbohydrates, and several major micronutrients. Although lentils are rich in inherent iron and zinc, they also contain certain antinutrient compounds, which may impede the uptake of these two minerals in the diet [4]. Enhancing the levels and bioavailability of these micronutrients through sustainable methods is a key research focus to ensure sufficient micronutrient intake and address micronutrient deficiencies [7].

**Figure 1:**
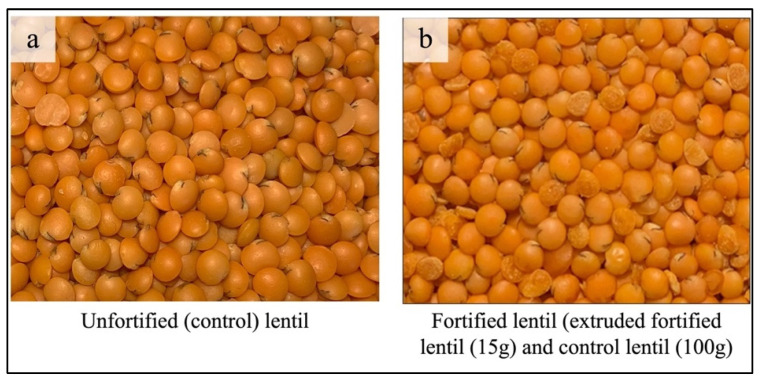
Pictures of uncooked lentil samples of two red lentil products: (**a**) unfortified control lentils and (**b**) multiple-micronutrient-fortified (MMF) lentils containing 15 g of extruded MMF lentil kernels mixed with 100 g of unfortified control lentils.

Various strategies are being implemented worldwide to tackle micronutrient deficiencies. These strategies include promoting dietary diversity, food fortification, biofortification, supplementation, public health interventions, disease control measures, nutrition education, and food safety initiatives. The goal of these efforts is to enhance the micronutrient content in crops and food products [8,9], and they have been implemented for a variety of staple foods and crops globally [10]. Among all approaches, fortifying staple foods is currently the most widely used strategy, with a proven history of enhancing dietary diversity and effectively reducing micronutrient deficiencies [9,10,11,12]. Around 125, 91, 32, 19, 12, and 7 countries have mandatory food fortification legislations for salt, wheat flour, vegetable oil, maize flour, sugar, and rice, respectively [13]. The Department of Plant Sciences of the University of Saskatchewan has been developing techniques and expertise in lentil fortification since 2014. A protocol was developed to fortify dehulled lentils using a spray application for delivering appropriate doses of Fe and Zn. This protocol aims to establish a strategy for lentil fortification and explore methods to increase Fe and Zn bioavailability in the human diet [7,14]. To add additional essential micronutrients, Hot Extrusion Technology was used to expand the essential micronutrient spectrum for lentil fortification to its full nutritional potential. In brief, extrusion trials were conducted using a co-rotating twin-screw extruder (Clextral EV-32, Firminy, France) fitted with a volumetric dispenser (Clextral VF/40/25-2) at the Saskatchewan Food Industry Development Centre Inc. (Saskatoon, SK, Canada). A micronutrient premix containing vitamins A, D3, B1, B3, B6, B9, and B12, along with the minerals iron (Fe) and zinc (Zn), was blended with lentil flour, emulsifiers, water, and steam to form a dough. This dough was extruded through a 2 × 4.25 mm conical die and then cut and dried to produce stable MMF lentil-shaped dal. The extruded lentils were dried using a steam dryer, and the moisture content was monitored with a moisture meter to maintain the product’s moisture at 10–12%. The selected extruded MMF lentils were blended with the dehulled control red lentils at a 15:100 ratio (MMF extruded/dehulled control lentils), and this blended lentil is considered MMF lentil (Figure 1). In a comprehensive approach, the total nutritional profile, protein digestibility, colorimetric changes, and stability of micronutrients in MMF lentils were assessed after six and twelve months of storage under retail market environment conditions [15]. This research intended to carry out a preliminary sensory assessment of MMF lentils by lentil consumers in Bangladesh. Sensory analysis is a multidisciplinary field encompassing various social sciences, including food science, statistics, and psychology. It records unbiased human reactions to food, helping stakeholders recognize the impact of brand [16,17]. Sensory tests fall into two main categories: analytical tests, which focus on the product, and affective tests, which focus on the consumer [18]. Exploring how fortification might change these attributes is an intriguing research area as it could affect consumer acceptance. The palatability of enriched foods relies on the kind and quantity of the fortificant, the desired food’s composition, and the chemical reactions among various fortificants [19]. Fortification may lead to an unpleasant metal-like flavor in foods due to lipid spoilage, unwanted color changes, and a reduction in the vitamins’ quality (for example, β-carotene and ascorbic acid have a significant influence on iron uptake and use) [20]. Every fortification initiative strives to minimize undesirable alterations in food or food products. A previous study evaluating consumer preferences for uncooked and cooked Fe-enriched lentils found that consumers preferred the Fe-enriched lentils over the control lentils [21]. The current study expects that MMF lentils will be just as acceptable to consumers regarding taste, smell, appearance, texture, and overall acceptability. Fortifying lentils with multiple micronutrients significantly affects preferences for their sensory qualities. Furthermore, there may be noticeable differences in sensory attributes between MMF and control lentils. Recognizing these sensory variations could have significant scientific consequences for the food science sector. Consumer feedback, an important source of insights, is essential in shaping guidelines for food researchers and industrial food processors. Additionally, understanding sensory acceptability can help determine the optimal baseline and limits for fortificant formulas to meet the recommended daily allowance of added micronutrients.

## 2. Materials and Methods

### 2.1. Study Design and Panelist Selection

A cross-sectional study was conducted from February to May 2024 at the Pulses Research Centre in Ishurdi, Bangladesh. The local investigator distributed a recruitment poster within the study area to notify potential participants about this study. The poster outlined inclusion and exclusion criteria, briefly described participant tasks, and estimated the time commitment required. Using a simple random sampling technique, participants from diverse age groups, genders, and socioeconomic backgrounds were recruited to represent the broader population of lentil consumers. Participants aged 16–65 years were included based on their willingness to participate and their overall health. The exclusion standards were (i) experiencing a viral or influenza infection or gingivitis; (ii) taking medications for any life-threatening diseases, such as malignancy, hyper or hypothyroidism, and neurological or psychoactive therapy; (iii) having a tendency of sensitive reactions to legumes or any vitamins or minerals added to fortify the lentils; (iv) gestation; and (v) consuming any mouth refresher or taste stimulator, such as “paan/jarda” (locally popular viny plant leaves and dried tobacco leaves) an hour prior to the sensory assessment. Trained research assistants conducted in-person interviews with participants in a private setting. A total of 200 untrained lentil consumers participated in the sensory assessment, even though the Institute of Food Technologists’ Sensory Evaluation Data guidelines suggest 50–100 responses for such evaluations [22].

### 2.2. Preperation of Uncooked and Cooked Lentils for Assessment

**Figure 2:**
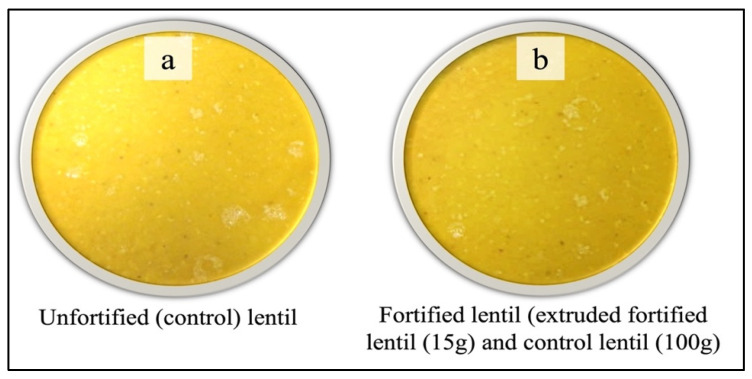
Images of cooked lentil samples of two red lentil products: (**a**) unfortified control lentils and (**b**) multiple-micronutrient-fortified (MMF) lentils containing 15 g of extruded fortified lentil kernels mixed with 100 g of unfortified control lentils.

A series of trials were conducted to choose the optimal micronutrient premixes at the Saskatchewan Food Industry Development Centre’s laboratory [15], and Canadian lentils were fortified with multiple micronutrients [i.e., vitamins A (retinol and β-carotene), B3 (niacin), B6 (pyridoxine), B1 (thiamine), B9 (folate), B12 (cyanocobalamin), and D3 and iron and zinc]. For the sensory evaluation, two uncooked samples (one MMF and one control lentil sample) were tested among the adult Bangladeshi consumers (Figure 1). Comparable uncooked lentils were used to prepare a popular traditional dish [23,24] frequently consumed in Bangladesh (Figure 2).

Both the control and MMF lentils were cooked on the data collection day at the Bangladesh Agricultural Research Institute (BARI) food processing laboratory in Ishurdi upazila, Pabna district, Bangladesh. Stainless steel cookware was used to ensure hygiene and quality during cooking. A semi-thick lentil soup was made using both the control and MMF lentils following a traditional recipe commonly used to cook lentils in Bangladesh. In brief, 1 kg of lentils from each of the control and MMF lentils was boiled with 2.5 liters of demineralized water for approximately 20 min. After boiling the lentils, 10 g dry turmeric powder, 20 g table salt, 30 mL canola oil, and 100 g chopped onion were mixed uniformly with an electric hand mixer. The uncooked MMF and control lentils were put into two 4-ounce white styrofoam cups, separately, and marked using 3-digit numbers for individual participant assessment. Following the evaluation of the uncooked lentils, the same participant received the cooked MMF and control lentil soup in two separate 3-ounce soup serving bowls marked with 3-digit numbers. Participants were provided with water to rinse their mouths before and after tasting each dish.

### 2.3. Instruments and Methods for Data Collection

Data collection was conducted in two phases. In the first phase (screening), participants were selected using probability sampling. Initially, 200 interested people (working-age populations, aged 16–65 years) who had experience with lentil consuming, growing, or marketing were enrolled to evaluate their socioeconomic characteristics using a screening form. Among the 200 individuals, 150 individuals were chosen for the sensory assessment. In the second phase, a different organized sensory data assessment form was utilized for sensory assessment. The screening and sensory data collection forms were translated back and forth between the Bangla and English languages. The sensory data assessment form comprised three sections. The 1st section gathered socioeconomic data, while the 2nd section evaluated the preferences for the five sensory attributes, namely the appearance, odor, taste, texture, and overall acceptability of the MMF lentils. A 9-point hedonic scale (1 = extremely dislike; 9 = extremely like) was deployed to determine the preference of both uncooked and cooked MMF lentils. The 3rd section captured any opinions or comments from participants about the tested samples verbatim, whether positive or negative. Participants received a lump sum amount (BTD ~500/USD 5.5) to compensate for their time and transportation to and from the study location.

The sensory evaluation took place over a day, from late morning to late afternoon. Fifteen Scientific staff members (SAs) from the Pulses Research Centre were hired the day prior to the sensory assessment and instructed by the project investigators from Bangladesh and the University of Saskatchewan. The instructions covered the survey procedure and insights of the sensory data collection form to ensure adherence to the questionnaire structure, queries, and response preferences, thereby minimizing interviewer bias. The questionnaire was pre-tested by the trained interviewers before the actual data collection process. Fifteen MMF lentil booths with uniform white lighting and furniture were set up for participants to test the sensory attributes. Each participant evaluated the control and MMF lentils while seated one-on-one with a scientific staff because the assistants were trained and explained the samples to the participants. Also, some of the participants needed help reading the consent form, questionnaire, and data collection form. Fifteen participants scored the uncooked and cooked lentils consecutively and in individual sessions to prevent selective reporting. At the beginning, two uncooked lentil samples were displayed on a white plastic tray for assessment. Then, the same participant received the cooked lentil soup including both the control and MMF sample in a separate soup bowl. Every participant rinsed their mouth with deionized water before testing the first cooked sample and following the test of the first sample before testing the second sample [23,25].

### 2.4. Ethical Approvals

Ethical approval to conduct this study was received from the Human Research Ethics Office at the University of Saskatchewan, Canada (Beh ID 3803). The second approval was received from the Institutional Review Board/Ethics Review Committee, North South University, Bangladesh (2023/OR-NSU/IRB/0205).

### 2.5. CIELAB Scores of Control and MMF Uncooked Lentils and Their Correlation with Sensory Traits

The initial colors (CIELAB color score, L*, a* and b*) of the control and MMF lentils were measured using a HunterLab (Hunter Associates Laboratory Inc., Reston, VA, USA) instrument. The Hunterlab L*, a*, and b* scales were measured four times per sample. L* represents the range from darkness to lightness, from 0 to 100; a* represents greenness to redness, from −80 to +80; and b* represents the range from blueness to yellowness, from −80 to +80 [26]. The Pearson correlation test was used to assess the correlation between sensory data for two traits (appearance and overall acceptability) of two uncooked MMF lentils and their L*, a*, and b* scores.

### 2.6. Statistical Analysis

**Figure 3:**
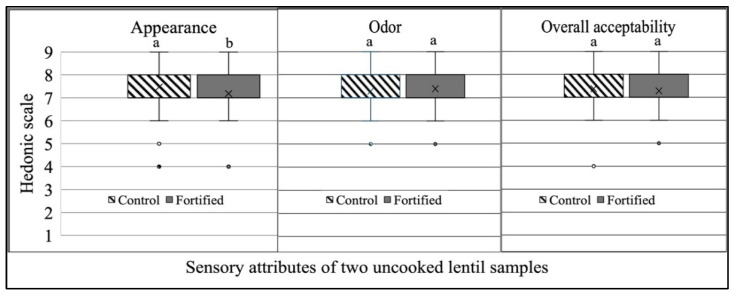
Box and whisker plot assessment of sensory attribute data (1 = extremely dislike; 9 = extremely like) obtained for control and multiple-micronutrient-fortified (MMF) lentils evaluated for three attributes (appearance, odor, and overall acceptability) by 150 panelists in Bangladesh. Distinct letters above box plots reveal significant variances among control and MMF lentils for all traits. Every box plot illustrates one sample’s (either control or MMF lentils) data dispersion individually using quintuple overview: minimum, first quartile (Q1), median, third quartile (Q3), and maximum.

**Figure 4:**
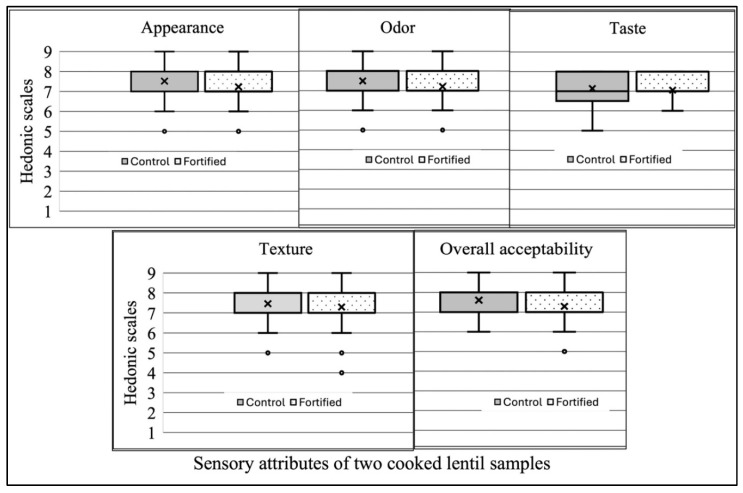
Box and whisker plot assessment of sensory attribute data (1 = extremely dislike; 9 = extremely like) obtained for control and multiple-micronutrient-fortified lentils. These lentils were evaluated for five attributes (appearance, odor, taste, texture, and overall acceptability) by 150 panelists in Bangladesh. Distinct letters above box plots indicate significant differences between two samples for each trait. Each box plot shows data distribution for each sample type individually using quintuple overview: minimum, first quartile (Q1), median, third quartile (Q3), and maximum.

Upon the completion of data collection, data were compiled using SAS (Statistical Analysis Software, SAS Institute Inc., Cary, NC, USA) version 9.4. The preliminary data collected from the 200 participants were examined by entering the first screening form’s information to test feasibility and ensure all variables stated in the screening form were included. Sensory scores for all the five traits of the control and MMF lentils were presented in a box plot (Figure 3 and Figure 4). A paired t-test was conducted at a 95% confidence interval to estimate the mean score contrasts between the control and MMF lentils’ sensory ratings obtained from the panelists. The CIELAB color scores were evaluated with R Core Team (2021) (R: A language and environment for statistical computing. R Foundation for Statistical Computing, Vienna, Austria. URL https://www.R-project.org/ (accessed on 5 August 2024)). Cronbach’s alpha (CA) coefficient was employed to evaluate the reliability of the participant’s feedback as it measures the “Internal Consistency Reliability” (ICR) of an evaluation panel in cumulative assessment scores [27]. The ICR was also used to evaluate the observational discrepancy (ranging from 0 to 1) by taking the square of the correlation (alpha values) and then deducting the outcomes from one, which revealed the variation in the observational discrepancy [28,29,30]. Although there is no strict threshold for Cronbach’s Alpha (CA), several studies indicate that acceptable internal consistency reliability varies from 0.70 to 0.95 [31,32].

## 3. Results

### 3.1. The Socio-Demographic Characteristics of the Participants

Table 1 provides a summary of the participants’ socio-demographic characteristics. Of the participants, the ratio of males to females was 1:1. Their ages ranged from 16 to 65 years, with the largest age group (33.3%) being 26–35 years old. The highest number of participants (76.0%) came from households with one to five employed individuals. Nearly one-half (55.0%) of the participants reported monthly earnings ranging from USD 85 to 168 (approximately BDT 10,000 and 19,999). The highest level of education attained by 43.6% of the participants was elementary (grade 5), while 27.5% had completed secondary education (grade 12).

### 3.2. Consumer Perspectives on Lentil Consumption

In this study, the highest number of individuals (57.3%) reported buying between 251 and 500 g of lentils each week, while 12.0% purchased between 751 and 1000 g, as detailed in Table 2. Additionally, these participants acquired smaller amounts of other types of pulses—such as chickpeas, mung beans, black gram, and field peas—with 51.3% buying 100–250 g and 37.3% obtaining 251–500 g on a weekly basis. The predominant place for purchasing lentils was local markets (94.0%), with neighborhood grocery stores accounting for a small portion (5.3%). Most panelists (85.3%) bought lentils monthly and showed a strong preference for red football lentil products (95.3%), while a minority (7.0%) chose red split lentil products.

### 3.3. Preference Towards Uncooked MMF Lentils

Figure 3’s box plots illustrate the range, average, distribution, and outliers of the appearance, odor, and overall acceptability of the uncooked control and MMF lentils. Consumer feedback showed notable differences in appearance but was consistent for odor and overall acceptability. The control and fortified lentils had similar range and dispersion.

### 3.4. Preference Towards Cooked MMF Lentils

Figure 4’s box plots illustrate the range, average, distribution, and outliers of the five sensory attributes (appearance, odor, taste, texture, and overall acceptability) for the two cooked samples (control and MMF lentils). Consumer responses were significantly similar for all five attributes, including overall acceptability. The control and MMF lentils had similar range and dispersion for all attributes except taste.

### 3.5. CIELAB Scores [L (Luniosity), a* (Redness), and b* (Yellowness)] of Uncooked Control and MMF Lentils and Their Correlation with Sensory Traits

Table 3 presents the changes in the CIELAB scores (L, a*, and b*) for both the control and MMF lentils. The CIELAB scores from the Hunter Lab measurements indicate insignificant variations in the a* and b* values between the control and MMF lentils. The ranges for the L, a*, and b* values in the control and MMF lentils were 55.62–58.07, 34.62–33.65, and 40.56–40.39, respectively. While the L value increased significantly, the a* and b* values remained significantly similar at the initial stage.

Table 4 shows a significant (*p* < 0.0001) correlation coefficient between the CIELAB color (L*, a*, and b*) scores from colorimetric measurements and ratings from two sensory traits (appearance and overall acceptability), with values varying from −0.98 to 0.97. A negative correlation between the L values and sensory scores was observed. Luminosity was increased significantly in the MMF lentils by adding a micronutrient premix. However, the consumers gave lower scores to the MMF lentils than the control ones. On the other hand, a positive correlation was observed between the sensory scores and red and yellow hue scores.

### 3.6. Evaluating the Reliability of the Sensory Ratings Using Cronbach’s Alpha

Cronbach’s alpha (CA) was employed to assess the sensory data reliability that measures the ‘proximity between evaluation profiles’ with regard to the associations between variance and covariance [27]. The reliability assessment shows the CA ratings for the cooked and uncooked control and MMF lentils (Table 5). For the uncooked lentils, CA was ≥0.70. The CA rating for the cooked control and MMF lentils was 0.79. The average CA ratings for the uncooked and cooked lentils were 0.71 and 0.79, respectively, representing strong uniformity in the sample evaluations by the consumers.

## 4. Discussion

In this study, the key sensory traits (i.e., appearance, odor, texture, taste, and overall acceptability) were compared for both cooked and uncooked dehulled red lentils fortified with multiple micronutrients and unfortified (control) lentils among Bangladeshi adults. The overall results show that appearance, taste, odor, texture, and overall acceptability did not significantly vary between the cooked MMF and control lentils. This suggests that the fortification process had a minimal or no impact on the sensory qualities of the MMF lentils, reinforcing the positive consumer response to the MMF lentils. We expected such findings because of the hot extrusion fortification technique (HET) that was used to fortify the lentils with multiple micronutrients. HET has been considered the most robust fortification technique, particularly when adding multiple micronutrients, since it can contain multiple micronutrients in a single food vehicle. Additionally, it allows the inclusion of by-products within the HET process, resulting in a financially viable product.

We added several vitamins, such as Vit A (retinol and beta-carotene), Vit B1 (thiamine), Vit B12 (cyanocobalamin), Vit B6 (pyridoxine hydrochloride), Vit B3 (niacin), Vit B9 (folate), and Vit D3, and micronutrients such as iron (Fe) and zinc (Zn), to fortify the MMF lentils. Vitamin and mineral deficiencies affect more than a third of the world’s population. To fight the consequences of such deficiencies, relevant staple foods are enriched with micronutrients. Lentils are one of them. Collectively, over 50 countries produce approximately 7.6 million tonnes of lentils globally, with Canada contributing about 50% (3.7 million tonnes) [3]. Some countries that do not produce lentils rely on imports to use them as a staple food. Lentils, a cost-effective solution, are favored in everyday meals due to their quick cooking time and affordability as a source of several micro and macro nutrients compared to animal-derived sources [33]. Lentils add complementary protein to rice-based diets, and most importantly, unlike rice, which in many countries is boiled in water which is then drained from the rice, cooked lentils are consumed as a soup without draining the cooking water, and this results in maximum nutrient availability after cooking in comparison to rice [7].

Fortifying lentils may be much more efficient because the volume of food products needed to achieve effective nutrition will be reduced. Many countries now accept extruded fortified rice to combat micronutrient deficiencies. Our previous study [15] proposed a similar approach to fortify lentils with multiple essential micronutrients. The extrusion techniques used for MMF lentils were similar to rice fortification, entirely different from the flour fortification technique used for maize and wheat. Finally, a novel method for fortifying lentils with multiple vitamins and minerals (vitamins A (retinol and beta-carotene), B1 (thiamine), B3 (niacin), B6 (pyridoxine), B9 (folate), B12 (cyanocobalamin), and D3; calcium, iron, and zinc) was developed using Hot Extrusion Technology (HET) [15]. These MMF lentils have been named multiple-micronutrient-fortified (MMF) lentils, and the MMF development protocol can easily merge with existing milling practices in the pulse industry.

A sensory evaluation measures consumer responses regarding their affinity, fondness, and acceptance of food or food stuffs [34]. In this study, we assessed the sensory traits of newly developed MMF lentils among Bangladeshi consumers in Bangladesh. This study’s overall findings show that the average scores for all the attributes of MMF lentils were not significantly different from those of the control lentils, except for appearance. This suggests that the fortification process had minimal or no impact on the sensory qualities of the MMF lentil sample, reinforcing the positive consumer response to MMF lentils.

In this study, Bangladesh was chosen as the research site for several causes. A number of countries consume lentils as a staple or semi-staple food, with 56% of the world’s lentils being consumed in Asia [17], particularly in Bangladesh. This study took place in a key lentil-growing region of Bangladesh, where farmers have extensive experience in cultivating, post-harvest milling, and promoting lentils. The Pulses Research Centre of Bangladesh is also situated in this area. Numerous domestic and global institutes are engaged in the Bangladesh healthcare sector, performing research, sensory assessments, and field studies with fortified foods, including fortified rice. Meals such as “daal (pulses) and vhat (rice)” (also known as “hotchpotch”), prepared with rice and dehulled lentils, are widely enjoyed and prevalent in most South Asian countries. Approximately 60% and 12% of Bangladeshi females eat dishes or meals including lentils three and four days a week, respectively [34]. Over 80% of the lentils in Bangladesh are imported from countries like Australia and Canada, offering a substantial opportunity to address micronutrient deficiencies in Bangladesh.

Moreover, food fortification is gaining momentum in Bangladesh, though the market currently offers limited options, with some products still under review. Presently, two fortified foods—vitamin A-enriched vegetable oil and iodine-fortified salt—are accessible to consumers [35]. Research is ongoing for other fortified foods, such as lentils, wheat flour, sugar, and rice [35]. A feasibility study on iron-fortified lentils with adolescent girls demonstrated positive acceptance [36]. Additionally, an extensive, double-blind, locally focused, randomly selected regulated study involving around 1200 adolescent girls demonstrated that iron-fortified lentils significantly enhanced their iron status [36].

In sensory evaluation studies, consumers are essential for assessing product differences and characteristics [37]. The required number of respondents for a consumer test varies based on the specific foods or food products being evaluated and the test’s purpose, duration, and cost [38]. In this study, we had an acceptable sample size (*n* = 150) to determine the sensory attributes, as previous studies recommend a range of 50–300 respondents for consumer acceptability tests [39]. The Institute of Food Technologists’ “Sensory Evaluation Data Guidelines” also suggested 50–100 responses for such evaluations for consumer-level research [22]. 

In a consumer-level sensory analysis, socio-demographic data are crucial for determining whether the participants accurately represent a large number of consumers when evaluating a particular food product. Previous research has shown that sociocultural diversity, demographic variables, and financial status influence consumer choices about food or food products [40]. In the current study, the socio-demographic data of the participants regarding lentil consumption highlighted the general consumer sample’s representativeness. Consumer preferences for lentil consumption in Bangladesh indicate a preference for red lentils over other legumes. Most of the participants (95.3%) favored the red football product type over the red split product type. Approximately 94% of consumers in the Ishurdi area buy lentils locally, where they are sold in open bags or in 1–2 kg paper/plastic bags. An earlier study [14] revealed that out of 196 consumers, 73 urban consumers purchased lentils from local markets or retail shops. Consumers also considered the MMF lentils as a premium food product that should be packaged in airtight and secured bags to maintain their grade and minimize adulteration possibilities.

Consumer responses were significantly not different between the two uncooked samples (MMF and control lentils) for all attributes except appearance. The average sensory scores for appearance, odor, and overall acceptability for the uncooked control and MMF lentils sample were 7.49 and 7.22; 7.31 and 7.43; and 7.39 and 7.32, respectively. Although the difference in appearance was significant, it was numerically very low. This could be due to the addition of only 15% of the extruded MMF lentils, which look similar to the control lentils. Overall, 40%, 34%, and 26% of the consumers reported lower, higher, and similar scores for the MMF lentils compared to the uncooked control lentils. These results suggest that multiple micronutrient fortification did not negatively impact the sensory characteristics of the MMF lentils. Similarly to the uncooked samples, the consumer responses to both cooked control and MMF lentils were significantly similar across all five attributes, including overall acceptability. The control and MMF lentils exhibited a similar range and dispersion for all attributes except taste. The average sensory scores of appearance, odor, taste, texture, and overall acceptability for the cooked control and MMF lentils were 7.54 and 7.44; 7.68 and 7.28; 7.36 and 7.14; 7.48 and 7.31; and 7.61 and 7.50, respectively. For both lentil product types, the MMF lentils scored lower on all five attributes than the control lentils. Overall, 55%, 24%, and 22% of the consumers reported lower, higher, and similar scores for the MMF lentils compared to the cooked control lentils.

The boxplots (Figure 3 and Figure 4) show some outliers for both the uncooked and cooked lentils, which might have significantly affected the mean hedonic scores of the respective lentil samples. A few consumers rated both the uncooked and cooked control and MMF lentils with lower hedonic scores (where a score of four indicates slightly dislike, and a score of five indicates neither dislike nor like). Overall, the liking scores indicated that both the cooked and uncooked lentils were equally accepted by the participants.

The consumer evaluations indicated no significant difference in preference for sensory qualities or overall satisfaction between the uncooked and cooked lentils. This uniformity in scoring could be attributed to cooking methods based on traditional recipes for lentil soup (Kohinoor et al., 2010) [24]. Fresh onion (*Allium cepa* L.) and dry turmeric (*Curcuma longa* L.) powder, which are the two key ingredients of the recipe as they are commonly used when cooking lentils, can enhance visual appeal by reducing darkness with turmeric’s yellow hue. They also modify the scent and flavor profiles, potentially masking any metallic taste from MMF lentils [23]. Similar negligible variances were observed when comparing the sensory characteristics of cooked conventional versus fortified rice [41]. Furthermore, this research utilized MMF lentils processed at the SFIDC, where 0.5–1% canola oil is typically employed during post-dehulling for an enhanced glossy appearance with the aim of increasing consumer appeal; however, other oils like palm or soybean oil were deliberately excluded from this process to prevent any potential impact on flavor or aroma. Participants were briefed on the recipe prior to commencing with sensory assessments.

A sensory analysis enables the rapid and cost-effective evaluation of products by representative consumers who regularly consume the food or food products and possess sensory abilities [42]. Fortification’s effect on the sensory traits of enriched foods varies greatly and depends on the components of the fortificants (minerals and vitamins), their doses, and the other ingredients required to develop the food or food products [19]. In this research, even though participants could readily distinguish the MMF lentils from the control sample, the overall acceptability was statistically comparable for both uncooked and cooked lentils.

The reliability of colorimetric ratings was assessed using Cronbach’s alpha (CA), showing that panelists consistently rated both uncooked and cooked lentils, with the CA values falling within the satisfactory score that varies between 0.75 and 0.95 [31,32]. However, the uncooked MMF lentils had a CA value of 0.68, which falls below the recommended range. This inconsistency might be due to the differing scores given for these lentils. One study observed that missing values can impact the psychometric properties of any test [43]. Since the data were cross-sectional, generalizability cannot be inferred from this study, so caution is advised when interpreting these specific results.

Lentils are often combined with rice as a staple food in many countries, such as Bangladesh. They are also a promising option for quick-cooking meals that can be further enhanced nutritionally. MMF lentils offer a sustainable whole food solution to address micronutrient deficiencies in countries where lentils are commonly consumed. A 50 g serving of MMF lentils in cooked lentils can provide approximately 992 IU of vitamin A palmitate, 0.06 mg of beta carotene, 93.8 IU of vitamin D3, 0.77 µg of vitamin B12, 1.22 mg of vitamin B6 (pyridoxine hydrochloride), 4.6 mg of niacinamide, 1.98 mg of thiamine mononitrate, 0.15 mg of folic acid, 11.2 mg of iron (ferric pyrophosphate hydrate), and 4.87 mg of zinc (zinc sulfate monohydrate). These amounts cover a significant portion of the Recommended Daily Amounts (RDAs) for these micronutrients, as advised by the World Health Organization (WHO).

## 5. Conclusions

Lentils fortified with multiple micronutrients (including vitamins A, B1, B3, B6, B9, B12, and D3 and iron and zinc)] using Hot Extrusion Technology showed indifferent sensory attributes for all attributes except for appearance compared to the unfortified Canadian (control) lentils.

## Figures and Tables

**Table 1:** Socio-demographic traits of participants involved in sensory evaluation study of multiple-micronutrient-fortified lentils conducted in Bangladesh.

Characteristics of Profile		Number (%)
Biological sex	Male	76 (50.7)
	Female	74 (49.3)
Age (years)	16–25	32 (21.3)
	26–35	50 (33.3)
	36–45	40 (26.7)
	46–55	19 (12.7)
	56–65	9 (6.0)
Count of employed individuals within household	1–5	114 (76.0)
	6–10	34 (22.7)
	≥11	2 (1.3)
Aggregate monthly earnings from various sources (Bangladeshi Taka)	5000–9999 (USD ~42 to 84)	11 (7.4)
	10,000–19,999 (USD ~85 to 168)	82 (55.0)
	20,000–29,999 (USD ~169 to 253)	28 (18.0)
	30,000–39,999 (USD ~254 to 337)	19 (12.8)
	≥40,000 (USD ≥338)	9 (6.0)
Education	Illiterate	2 (1.3)
	Did not complete elementary (primary; grade 5)	7 (4.7)
	Completed elementary	65 (43.6)
	Received secondary school certificate (grade 10)	34 (22.8)
	Received secondary certificate (grade 12)	41 (27.5)

**Table 2:** Lentil consumption behaviors and trends among consumers.

Perception of Study	Consumers’ Pulse Buying Trends (g/Family/Week)	Consumer Numbers (%)
Lentils purchased by consumers	100–250	24 (16.0)
	251–500	86 (57.3)
	501–750	13 (8.7)
	751–1000	18 (12.0)
	≥1001	9 (6.0)
Other pulse (Bengal gram, Vigna radiata, Vigna mungo, pisum, etc.) purchased by consumers	100–250	77 (51.3)
	251–500	56 (37.3)
	501–750	5 (3.3)
	751–1000	3 (2.0)
	≥1001	9 (6.0)
Lentil purchase sources	Retail shops	141 (94.0)
	Wholesale	8 (5.3)
	Do not buy or produce	1 (0.7)
Consumers’ lentil-buying frequencies	Several days in a week	7 (7.4)
	Weekly	11 (7.3)
	Fortnightly	4 (2.7)
	Monthly	128 (85.3)
Consumers’ preferences when purchasing lentil products	Dehulled football	143 (95.3)
	Dehulled split	7 (4.7)

**Table 3:** CIELAB color (L*, a*, and b*) scores of control and MMF lentils.

	Lentil Samples	CIELAB Color Score [Mean (CI 95%)] ^a^
Luminosity (L*)		
	Control	55.62 ± 0.50 ^a^
	Multiple micronutrient fortified	58.07 ± 0.23 ^b^
Redness (a*)		
	Control	34.62 ± 0.16 ^a^
	Multiple micronutrient fortified	33.65 ± 0.35 ^a^
Yellowness (b*)		
	Control	40.56 ± 0.34 ^a^
	Multiple micronutrient fortified	40.39 ± 0.39 ^a^

^a^ Mean scores (CI 95%) for CIELAB color (L*, a*, and b* scores) followed by different Roman letters within two rows of each CIELAB color scores are significantly different (*p* < 0.001).

**Table 4:** Correlation coefficients between CIELAB color scores [luminosity (L*), red hue (a*), and yellow hue (b*)] obtained from colorimetric measurements and ratings of two sensory traits (appearance and overall acceptability) from 150 participants were analyzed for two uncooked lentils (control and multiple-micronutrient-fortified lentils). All correlation coefficients were determined to be significant at *p* < 0.0001.

Sensory Traits	CIELAB Color Scores Obtained from Colorimetric Mesurements Using Hunterlab		
	L	a*	b*
Appearance (*n* = 3)	−0.98	0.96	0.95
Overall acceptability (*n* = 3)	−0.98	0.97	0.96

L*, luminosity; a*, redness; b*, yellowness.

**Table 5:** Reliability assessment (Cronbach’s Alpha) score of participants’ sensory ratings for uncooked and cooked control and multiple-micronutrient-fortified (MMF) lentils.

Lentil Samples		Cronbach’s Alpha Score
Uncooked	Control	0.75
	Multiple micronutrient fortified	0.68
Uncooked sample’s average value		0.71
Cooked	Control	0.79
	Multiple micronutrient fortified	0.79
Cooked sample’s average value		0.79

## Data Availability

The data presented in this study are available upon request from the corresponding author due to institutional ethical policy.
